# Comparison of Two Different Modes of Active Recovery on Muscles Performance after Fatiguing Exercise in Mountain Canoeist and Football Players

**DOI:** 10.1371/journal.pone.0164216

**Published:** 2016-10-05

**Authors:** Anna Mika, Łukasz Oleksy, Renata Kielnar, Ewa Wodka-Natkaniec, Magdalena Twardowska, Kamil Kamiński, Zbigniew Małek

**Affiliations:** 1 Department of Clinical Rehabilitation, University of Physical Education in Krakow, Poland; 2 Oleksy Physiotherapy Clinic, Rzeszow, Poland; 3 Institute of Physiotherapy, Faculty of Medicine, University of Rzeszow, Rzeszow, Poland; 4 Department of Orthopaedics, Jagiellonian University, Collegium Medicum, Krakow, Poland; 5 Institute of Physical Education, State Higher Vocational School in Nowy Sącz, Nowy Sącz, Poland; 6 Physiotherapy Clinic „Effective Rehabilitation”, Warsaw, Poland; Universite de Nantes, FRANCE

## Abstract

**Background:**

The aim of this study is to assess if the application of different methods of active recovery (working the same or different muscle groups from those which were active during fatiguing exercise) results in significant differences in muscle performance and if the efficiency of the active recovery method is dependent upon the specific sport activity (training loads).

**Design:**

A parallel group non-blinded trial with repeated measurements.

**Methods:**

Thirteen mountain canoeists and twelve football players participated in this study. Measurements of the bioelectrical activity, torque, work and power of the vastus lateralis oblique, vastus medialis oblique, and rectus femoris muscles were performed during isokinetic tests at a velocity of 90°/s.

**Results:**

Active legs recovery in both groups was effective in reducing fatigue from evaluated muscles, where a significant decrease in fatigue index was observed. The muscles peak torque, work and power parameters did not change significantly after both modes of active recovery, but in both groups significant decrease was seen after passive recovery.

**Conclusions:**

We suggest that 20 minutes of post-exercise active recovery involving the same muscles that were active during the fatiguing exercise is more effective in fatigue recovery than active exercise using the muscles that were not involved in the exercise. Active arm exercises were less effective in both groups which indicates a lack of a relationship between the different training regimens and the part of the body which is principally used during training.

## Introduction

Exercise induced muscle fatigue is defined as a reversible loss of muscle force (muscle contractility) during work over time [1], and can last a few minutes, hours, or days. A wide range of recovery methods are now used as integral parts of the training programmes of elite athletes to help attain an optimal balance [2,3]. Therefore it is crucial to determine their efficacy and substantiate their use.

Some studies have explored recovery strategies including active recovery [4,5,6], but there are doubts about the influence of active recovery on subsequent performance, especially because in these studies there are methodological differences in relation to the task that is used as the performance criterion.

A number of methods exist to quantify neuromuscular fatigue in humans during muscular work [6–9]. Continuous monitoring of local muscle fatigue during performance is possible by measuring myoelectric activity of specific muscles using surface electromyography (sEMG). Its advantages are: noninvasiveness, real-time fatigue monitoring during the performance, which are correlated with biochemical and physiological changes in muscles during fatigue [10,11].

As has been postulated fatigue during dynamic exercise is accompanied by changes in electromyographic muscle activity which may be due to insufficient muscle blood supply [6,12]. The amplitude of the EMG signal during muscle activity initially increases and then, as the fatigue symptoms intensify, the value of the parameters decreases [10,13,14]. In the initial phase of fatigue on the peripheral level, a decline in the activity of motor units can be observed, which results in a gradual decrease in muscle contraction power [15]. To keep the muscle activity at the required level, the central nervous system increases central stimulation of the motor units. As a result, electrical discharge in active motor units occurs more often and more motor units are activated, including inactive units. This leads to an increase of the amplitude, and a higher value of the fatigue index, that is a higher slope of the regression line indicates higher levels of muscle fatigue [14,16,17]. Numerous studies have been conducted to compare the effects of active and passive recovery [18–21]. Present knowledge supports the superiority of active recovery methods over passive ones for removing lactate during exercise [18,20,21]. However, the effects of these recovery modes on subsequent performance are equivocal [22]. For instance, although some authors have reported that active recovery is more efficient than passive recovery [4,5], others found no differences [22,23], or a better physical performance after passive recovery [19,24]. As was assumed by some authors [22] the faster elimination rate of blood lactate concentration through active recovery is of no practical relevance for many disciplines and it may negatively affect the adaptation.

Additionally, it has been reported that post-exercise recovery should be active and should involve muscle groups which remained unaffected by fatigue [3,25]. Baker et al [25] also used a type of recovery focusing on different muscle groups than those utilized in the exercise, and suggested that this may optimize lowering blood lactate concentrations. However, some authors have indicated that the lower lactate concentration after active recovery did not cause a performance improvement [3,26,27]. Further studies using different types of recovery exercises are therefore needed to confirm this hypothesis and determine whether active recovery using a different muscle group can also improve performance.

The aim of this study was to examine the efficacy of two modes of post-exercise active recovery and passive recovery in reducing of fatigue. It also examined if the application of these recovery methods makes it possible to observe significant differences in the force and bioelectrical activity of the muscles tested.

The novel aspect of this study is the assessment of active recovery efficacy using the same and different muscles from those which were active during the fatiguing exercise. To differentiate the efficacy of the active recovery modes depending on the specifics of the training loads, the study group included mountain canoeists (who load mainly muscles of the upper body) and football players (who load mainly muscles of the lower body). The study also examined which form of active recovery is more effective in reducing of post-exercise muscles fatigue, and whether effectiveness of any one of these forms of active recovery is related to the specific training of the sporting discipline.

In this work for the first time we differentiate the effectiveness of the active arms and active legs recovery depending on the specifics of the discipline trained by athletes and which part of the body is mainly loaded during training.

## Materials and Methods

### Participants

**Fig 1:**
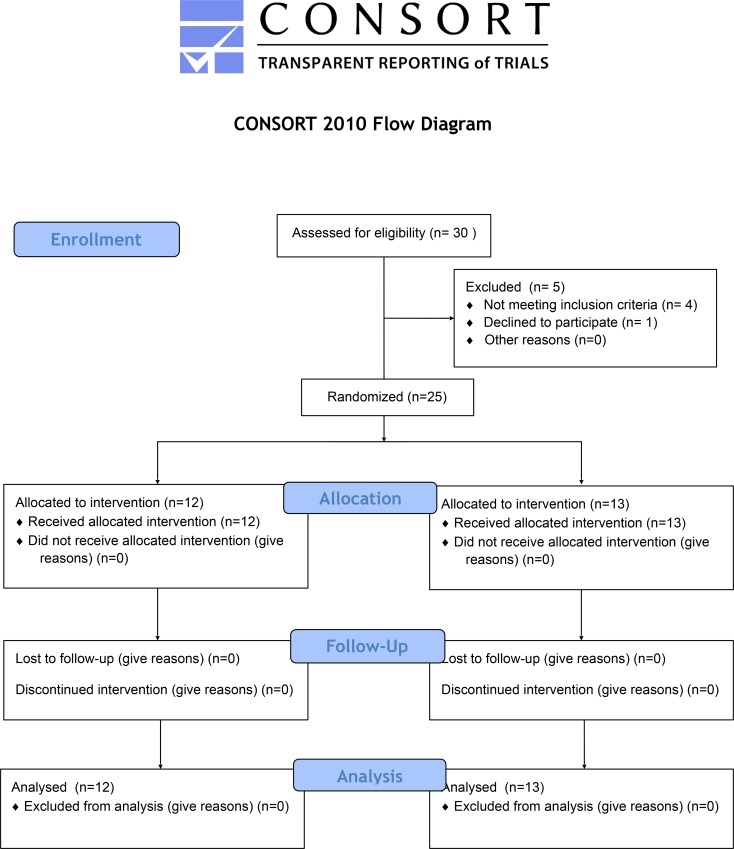
Consort diagram.

13 male mountain canoeists and 12 male football players (age 24–30 years old) participated in this study (Fig 1). The athletes belonged to a regional team, and all were healthy, with no injuries during the year before the study. They did not perform any high-intensity physical activity for 2 days before each visit to avoid the effects of cumulative muscular fatigue. The recruitment and follow-up of the study participants was performed at the biomechanical laboratory from September 2010 to July 2011. All measurements were performed by one examiner. Study participants were all informed in detail about the research protocol and gave their written informed consent to participate in the study. The Ethical Committee of Regional Medical Chamber in Krakow approval was obtained for this study. This study was registered in the Australian New Zealand Clinical Trials Registry (ANZCTR). Registration number: ACTRN12616000384459. The trial was registered retrospectively, because it did not include any drug or medical intervention. The kind of intervention (exercise) allow us to register the trial as ongoing study after the first participant enrollment. The data presented in the current study are a part of a wider project. The authors confirm that all ongoing and related trials for this intervention are registered.

### Procedures

#### Pre-test Visit

The participants visited the laboratory to become familiar with the protocol for the isokinetic testing. Measurements of body weight and height of the subjects were taken.

During this visit the maximum velocity of each subject’s treadmill running (Woodway, USA) at a tilt of 12% (TV 12%) was assessed to determine the intensity of the test physical effort (120% with TV 12%) [19]. The initial speed of the treadmill was 5 km/h and was increased 1 km/h every 2 minutes, and physical effort was performed to exhaustion, or to refusal to continue by the subject. The 100% it was the speed at the end of the effort when the subject was exhausted. From the maximal treadmill speed the 120% was calculated. The fatiguing exercise involved ten treadmill runs, each one-minute long (performed at the intensity of 120% of maximal treadmill speed determined during pre-test visit) interspaced with two-minute breaks. During each two-minute break, the treadmill was stopped and the subject rested by standing on the treadmill. Then the treadmill was started and accelerated to the 120% of maximal treadmill speed. The test physical effort lasted 30 minutes (10 one-minute runs and 10 two-minute breaks). If the subject was not able to complete the test at the required intensity, the velocity was reduced by 0.5 km/h.

#### Visit 1,2,3

The subsequent three visits evaluated three muscle recovery methods after intensive physical exercises.

Each session began with a warm-up, which consisted of cycling for 5 minutes (Keiser M3, Germany). The volunteers cycled at a comfortable, self-selected speed. All measurements were taken from the dominant side (right or left leg). Measurements of the bioelectrical activity (EMG) of the vastus lateralis oblique (VLO), the vastus medialis oblique (VMO), and the rectus femoris (RF) muscles and the torque, work and power of the knee flexor and extensor muscles were performed during isokinetic testing at a velocity 90°/s. The measurements were performed at 3 visits with one week intervals, and at each visit before fatiguing exercise, after exercising and after 20 minutes in one of the recovery modes (pedaling on the cycle ergometer, pedaling on the arm ergometer, or passive rest in a sitting position).

#### Force measurement

The measurement was taken using an isokinetic dynamometer (Biodex System S4, USA) in a sitting position with the lower extremity flexed in a hip joint to 90°, with the knee axis of rotation concordant with the anatomical axis of the joint. To prevent trunk movements during measurement, the subjects were fastened with a stabilizing strap [28]. The movable arm of the dynamometer was fixed at the distal end of the tibia, proximal to the medial malleoli. This position was recorded to ensure the same placement for all 3 test sessions. Gravity correction was obtained by measuring the torque exerted on the dynamometer resistance adapter by the relaxed, fully extended knee. Total range of motion (ROM) during the isokinetic contractions was set from full extension to full flexion. The tests consisted of 10 maximal isokinetic concentric knee extensions and flexions at an angular velocity of 90°/s [28].

As the dynamometer arm moved, the participant was verbally encouraged to perform maximally for each contraction throughout the full ROM during both the flexion and the extension phase. All the testing procedures and verbal encouragement were administered by the same investigator to all athletes. The torque, work and power of knee flexors and extensors were calculated as a mean value of 10 contractions. As was reported by Larsson et al. [29,30] the reliability of peak torque was good and ICC ranged between 0.85–0.98 for knee extension and 0.88–0.97 for knee flexion.

Torque and sEMG measurements were recorded simultaneously and continuously while the participants performed the 10 isokinetic knee extensions. As has been reported, the measurement of bioelectrical activity of VM, VL, RF during knee joint flexion and extension in the isokinetic condition at a velocity of 90/s has good repeatability (ICC > 0.8) [29,30].

#### The EMG measurement

The bioelectrical activity of the vastus lateralis (VL), the vastus medialis (VM), and the rectus femoris (RF) was recorded according to the SENIAM guidelines [31,32]. Prior to electrode placement the skin was cleaned and degreased with alcohol. Surface electrodes (Ag/AgCl) (BIO LEADLOK) with a 2 cm center-to-center distance were attached along the direction of the muscle fibers on the bellies of VL, VM, RF.

The signals were registered with 16-bit accuracy at a sampling rate of 1500 Hz and stored for subsequent analysis using Noraxon G2 TeleMyo 2400 unit (Noraxon USA). The EMG signals were filtered with a Butterworth high-pass filter (cutoff frequency 10 Hz) and a low-pass filter (cutoff frequency 500 Hz), and then rectified. Subsequently, the root mean squared (RMS) value of the EMG signal was determined over a 300-msec window. During the dynamic trials, approximately 10 peak values were calculated for each flexion-extension cycle for each muscle and used as data set for regression analysis. Calculated slope was used as fatigue index [16,17,32].

The values of the evaluated parameters obtained before and after physical effort, and after recovery were compared separately for each recovery method.

The measurement order was as follows:

1. Warm-up
2. Force and sEMG measurement before physical effort
3. Physical effort test—ten treadmill runs, each one-minute long and each performed at the intensity of 120% TV 12%, interspaced with two-minute breaks.
4. Force and sEMG measurement—taken immediately after the physical effort was completed (the measurement of the fatigue level)
5. Recovery–one of the three muscle recovery methods was applied immediately after the force and EMG measurements. The order of the 3 recovery methods was randomized.
  Active Legs Recovery (ALR)—pedaling on the cycle ergometer at a velocity of 60 rpm with a 10W load for 20 minutes (Keiser M3, Germany)
  Active Arms Recovery (AAR)—ride on the arm ergometer at a velocity of 60 rpm with a 10W load for 20 minutes (Sci-Fit, USA) (upper extremity work aimed at stimulating different muscle groups than those worked during the physical effort)
  Passive Recovery (P)–rest in a sitting position for 20 minutes
6. Force and sEMG measurement–taken immediately after recovery was completed (the evaluation of the recovery efficacy)

### Statistical Analysis

The statistical analysis was carried out using the STATISTICA 10.0 Pl. The ANOVA test with repeated measures was used to determine the significance of the differences of the evaluated variables. The independent t-test was used to evaluate the differences in body height and weight between mountain canoeist and football players. Differences were considered to be statistically significant if the level of the test similarities was lower than the assumed level of significance (p < 0,05). Additionally the data were tested for practical relevance using Cohen d effect size. A paired t-test power analysis of exercise influence determined that at least 9 subjects were required to obtain a power of 0.8 at a two-sided level of 0.05 with effect size d = 0.8. This analysis was based on data derived from previous literature [21,23,28,33].

## Results

There were no significant differences in body height and weight between mountain canoeists and football players (175.6 ± 3.8 vs. 179.3 ± 4.2 cm; 78.3 ± 6.49 vs. 75.8 ± 6.2 kg) (p>0.05).

### sEMG measurement

**Fig 2:**
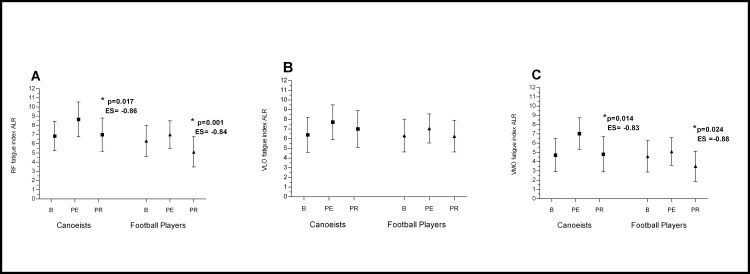
**Effects of Active Legs Recovery on RF (A), VLO (B), VMO (C) bioelectrical activity.** *p significantly different value; ES–effect size; ALR—Active Legs Recovery; RF- Rectus Femoris; VLO—Vastus Lateralis Oblique; VMO - Vastus Medialis Oblique; B—baseline; PE—post-execise; PR—post-recovery.

**Fig 3:**
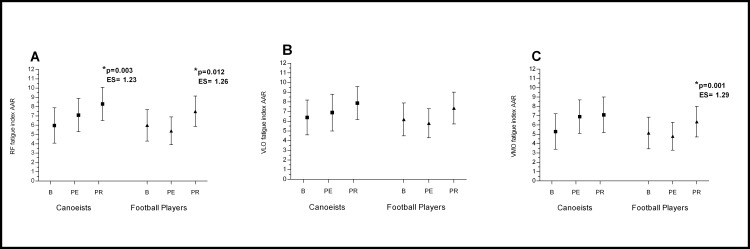
**Effects of Active Arms Recovery on RF (A), VLO (B), VMO (C) bioelectrical activity.** *p significantly different value; ES–effect size; AAR—Active Arms Recovery; RF- Rectus Femoris; VLO—Vastus Lateralis Oblique; VMO - Vastus Medialis Oblique; B—baseline; PE—post-execise; PR—post-recovery.

**Fig 4:**
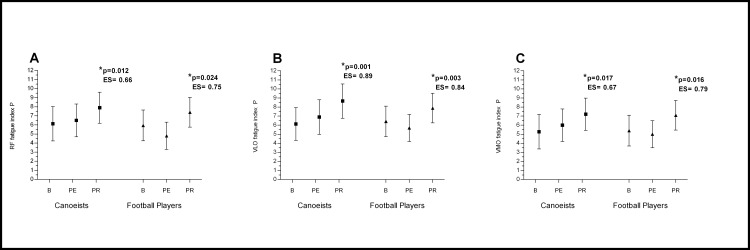
**Effects of Passive Recovery on RF (A), VLO (B), VMO (C) bioelectrical activity.** *p significantly different value; ES–effect size; P—Passive Recovery; RF- Rectus Femoris; VLO—Vastus Lateralis Oblique; VMO—Vastus Medialis Oblique; B—baseline; PE—post-execise; PR—post-recovery.

In mountain canoeists running on the treadmill resulted in higher fatigue seen as a higher sEMG fatigue index in comparison to baseline. In football players the same effort resulted in a lower fatigue index than in canoeists, but in both groups the changes after physical effort in comparison to baseline were non-significant (Figs 2 and 3 and 4) (p>0.05)

The evaluated muscles recovered sufficiently reaching their pre-exercise value in both groups only after active legs recovery (ALR), where a significant decrease in fatigue index compared to post-exercise value was observed in the RF and VMO muscles (Fig 2). After active arms recovery (AAR) a significant increase in the fatigue index in comparison to post-exercise value was noted in the RF in both groups, and in the VMO muscle in football players (Fig 3). A higher value of fatigue index was observed after passive rest in comparison to post-exercise and to baseline value. This significant increase was noted in both groups (Fig 4).

#### Force measurement

**Fig 5:**
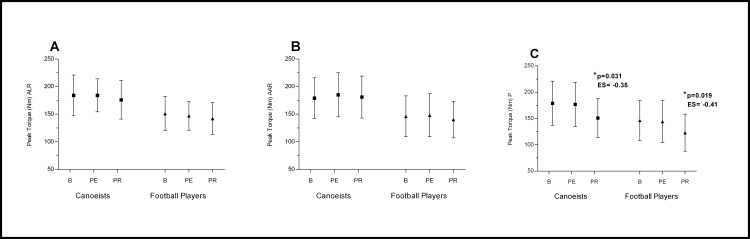
**Effects of ALR (A), AAR (B) and P (C) on evaluated muscles Peak Torque.** *p significantly different value; ES–effect size; ALR—Active Legs Recovery; AAR—Active Arms Recovery; P—Passive recovery; B—baseline; PE—post-execise; PR—post-recovery.

**Fig 6:**
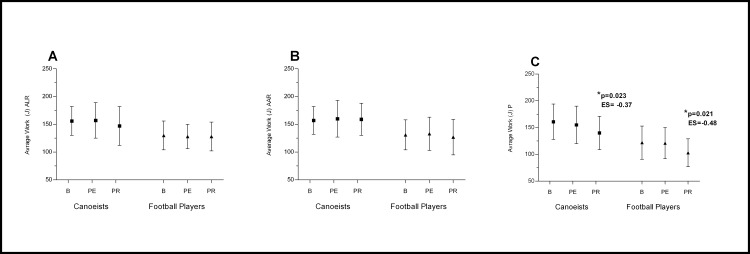
**Effects of ALR (A), AAR (B) and P (C) on evaluated muscles Average Work.** *p significantly different value; ES–effect size; ALR—Active Legs Recovery; AAR—Active Arms Recovery; P—Passive recovery; B—baseline; PE—post-execise; PR—post-recovery.

**Fig 7:**
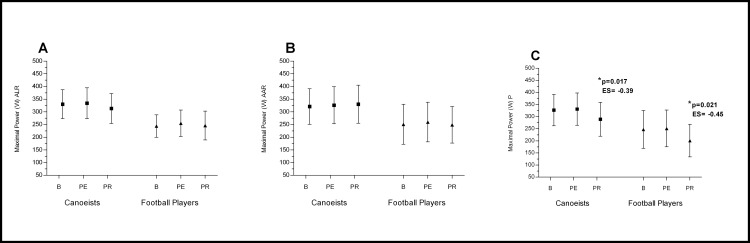
**Effects of ALR (A), AAR (B) and P (C) on evaluated muscles Maximal Power.** *p significantly different value; ES–effect size; ALR—Active Legs Recovery; AAR—Active Arms Recovery; P—Passive recovery; B—baseline; PE—post-execise; PR—post-recovery.

The changes in muscle force parameters were smaller than changes in muscles bioelectrical activity. (Figs 5 and 6 and 7)

There was no significant effect of active legs recovery (ALR) and active arms recovery (AAR) on muscle peak torque (PT) (Fig 5), work (AW)(Fig 6) and power (MP)(Fig 7) (p>0.05). Only after passive recovery (P) in both groups a significant decrease in PT, AW and MP was observed (Figs 5 and 6 and 7).

## Discussion

The most important information obtained in this study is the observation that during the post-exercise muscle recovery, mild active exercise of the same muscles used by the fatiguing exercise facilitate the change in the parameters of muscle bioelectrical activity to their pre-exercise value. Active exercise of the lower limbs was more effective in removing muscle fatigue in both evaluated groups (mountain canoeists and football players). Peak torque, work and power was decreased after passive recovery in both groups, whereas after active legs and active arms rest the value of these parameters was unchanged compared to baseline. In both groups independent of their sporting discipline training details, active legs exercise by cycling on a bike ergometer improved fatigue recovery more effectively than when active recovery was performed using an arm ergometer.

There is some evidence that active recovery can be a good strategy to improve performance [3,18,22,34]. In the present study, the decrease in the fatigue index after active legs recovery indicates that less motor units were activated during the exercise effort than after passive recovery. This may suggest that passive rest is not a recommended option for muscle recovery following fatiguing exercise. A significant decrease in peak torque, work and power after passive recovery was observed in both groups compared to baseline, and indicates an unfavorable influence of passive rest on muscle performance recovery after fatigue. After active legs and active arms recovery the value of the force related parameters was unchanged. The results from the present study are in agreement with previous research [3,18,22,35] that has demonstrated advantages of active muscle recovery. These results are in line with our previous study, where after active recovery the mean muscular voluntary contraction value was similar to baseline, but after passive rest was significantly decreased [33].

The higher value of the fatigue index and the decrease in torque, work and power after passive rest observed in our study may suggest that after physical effort passive recovery applied over 20 minutes does not remove the symptoms of muscle fatigue. The absence of differences in the fatigue index after active legs recovery compared to the baseline value may suggest that mild exercise applied after intensive physical effort may accelerate the removal of the muscle fatigue symptoms, and thus keep the potential for the recruitment of the appropriate amount of motor units during subsequent physical effort.

In contrast, some authors have reported that both active and passive recovery regimens have the same influence on the parameters obtained during subsequent physical effort [4,18,20,22,23]. The discrepancy between these reports may be caused both by a variety of control charts of the test physical effort used by the authors, and by the different lengths of recovery time examined.

As was suggested previously, active legs recovery may be considered a much more effective recovery process than massage and passive rest, particularly when a faster rate of lactate elimination is the main criterion [5,36,37]. The different criteria used for the recovery process evaluation employed in our study (muscle force and EMG analysis) support those metabolic observations.

It has been suggested that the intensity of the exercise during active recovery may be optimal for the rate of lactate elimination and phosphocreatine resynthesis [38]. Therefore the muscle motor units involved in the active recovery should be optimal. If the intensity of exercise is too high the energetic cost is high making the amount of oxygen too low for haemoglobin reoxygenation. This may explain why a significant improvement of performance (65–27%) was observed only in those studies where the intensity of active recovery was very low (28% VO2max) [38,39]. When the intensity of active recovery was higher (40% of VO2max), the improvement in performance was marginal [35]. Therefore some authors have suggested that the optimal level of muscle recovery may be obtained by the appropriate work intensity [38], or by the activity of different muscles groups which did not involve in fatiguing effort [3,25]. Baker et al [25] performed a type of recovery focusing on a different muscle group than that utilized in the exercise, which as they hypothesized, may optimize the lowering of the blood lactate concentration. Tiret et al [3] also assessed 16 healthy male gymnastic students using three recovery modes: active legs, active arms and passive recovery. But they concluded that further studies were needed to confirm the efficacy of different muscles groups exercise in post fatigue recovery and to determine whether active recovery using a different muscle groups can also improve performance.

Research in the same area found that if another muscle group is activated after a physical exercise, work performed after active recovery is greater than after passive recovery [28,40]. However, it was also demonstrated that when lactate was produced through exhaustive arm work, reduced intensity arm work during the recovery phase was less effective in removing lactate than leg exercise [25]. In this study, after 20 minutes of cycling with minimal resistance the lower limb muscle bioelectrical activity returned to near pre-exercise values indicating that this kind of active exercise removes fatigue and recovers the exercising muscles better than arm exercise or passive rest. The superiority of active recovery using the same muscles which were active during fatiguing exercise on recovery were reported in both groups–football players and mountain canoeists. Therefore, based on our observations we have suggested that the light active exercise is effective in removing post exercise muscles fatigue independently of the sport disciplines specific muscles training. In our study both the football players, who load in daily training mainly lower limbs muscles, and canoeists, in contrast load mainly the upper body muscles, reacted similarly to the recovery methods applied. Active arm exercise were less effective in both groups, which indicates a lack of relationship between the specifics of the discipline trained by the athletes (that is which part of the body is primarily loaded during training) and the efficacy of active recovery.

An important issue is the length of recovery time between muscular efforts. In the present study, 20 minutes of rest was applied. Lariviere et al. [12] used sEMG to evaluate passive rest intervals of 10 and 15 mins after fatiguing back exercise. Their results suggest that complete muscle recovery was achieved with 10- to 15-min rest periods. Vaz, et al. [41] evaluated the post-exercise shifts in the average frequency of the sEMG signal of the RF and VLO muscles, and observed that after 15 minutes of passive recovery the velocity of conduction of motor units regained its threshold value, but the amplitude was still higher than its pre-exercise value. Esposito et al. [42] proved that 10 minutes after passive recovery, post-exercise muscle fatigue caused a decrease in MVC by 26% and noticeable shifts in the amplitude and the frequency of the sEMG signal. The changes of these parameters occurred despite the fact that the level of force parameters obtained after recovery and before physical effort was the same. In this study similar results in the muscle sEMG activity and force parameters were obtained.

In the present study, the subjects ran on the treadmill to the point of fatigue, followed by a 20-minute recovery period. In our previous study [33] a shorter recovery time (5 minutes) was used, and this may be the reason for the significant decrease in MVC observed after recovery. On the basis of data from our previous study [33] and from other authors observations [12,43], short rest periods seem insufficient to allow full recovery and longer rest periods of 10–20 minutes would be more appropriate. After 20 minutes of cycling with minimal resistance, the lower limb muscle bioelectrical activity returned to pre-exercise values indicating that this kind of active exercise allows the removal of fatigue and recovery of the exercising muscles better than when using arm exercise or passive rest.

There are several limitations of this study that need to be addressed. First, the study population consisted of football players and mountain canoeists, so these findings may not be able to be extrapolated to other sport disciplines, and future research should be conducted with other groups of athletes. Additionally, the present study fatiguing protocol involved only running on the treadmill, affecting therefore only the lower limb muscles. We think that future studies should also include fatiguing exercise of the upper limbs. The use of two methods of the fatiguing protocol and two methods of active recovery (active arms and active legs) may allow the exploration of the post exercise recovery efficacy more comprehensively.

## Conclusions

The purpose of the present study was to investigate the effect of three recovery methods on muscle peak torque, work and power, and on muscle bioelectrical activity after exhaustive treadmill running with 20 minutes of post-exercise recovery.

The superiority of active recovery using the same muscles which were active during the fatiguing exercise on recovery were reported in both groups–football players and mountain canoeists. Therefore, based on our observations we have suggested that this kind of light active exercise is effective in removing post exercise muscles fatigue independently of sport discipline training specifics. In our study both the football players, who in daily training load mainly lower limb muscles, and canoeists, who in contrast load mainly upper body muscles, reacted similarly to the recovery methods applied. Active arm exercises were less effective in both groups which indicates a lack of a relationship between the specifics of the discipline trained by the athlete (and therefore which part of the body is mainly loaded during training) and the method of active recovery efficacy.

Based on our results we suggest that 20 minutes of post-exercise active recovery by working the same muscles that were active during the fatiguing exercise is more effective in fatigue reduction than active exercise using those muscles not involved in the fatiguing effort.

## Practical Implications

Based on our results we suggest that 20 minutes of post-exercise active recovery by working the same muscles that were active during the fatiguing exercise is more effective in fatigue removal than active exercise using those muscles not involved in the fatiguing effort or passive rest. This kind of light active exercise is effective in removing post exercise muscles fatigue independently of sport discipline training specifics.

## Supporting Information

S1 Checklist Trendstatement checklist. (PDF)

S1 Protocol Bioetical protocol of this study–English. (PDF)

S2 Protocol Bioetical protocol of this study–polish. (PDF)

## Abbreviations

AAR: Active arms recovery
ALR: Active legs recovery
AR: Active relaxation modes
AW: Average work
MVC: Maximal voluntary contraction
MP: Maximal power
P: Passive recovery
PT: Peak torque
RF: Rectus femoris
RMS: Root mean squared
ROM: Range of motion
sEMG: Surface electromyography
TV: Treadmill velocity
VLO: Vastus lateralis oblique
VMO: Vastus medialis oblique
