# Supplementary material for: Comparison of Two Different Modes of Active Recovery on Muscles Performance after Fatiguing Exercise in Mountain Canoeist and Football Players
Source: PLoS One. 2016 Oct 5;11(10):e0164216. doi: 10.1371/journal.pone.0164216 (PMC5051742; doi:10.1371/journal.pone.0164216)
Supplement: S1 Protocol — (PDF) [file pone.0164216.s002.pdf]

The Ethical Committee of Regional Medical Chamber in Krakow
ul. Krupnicza 11a,
31-123 Kraków, Poland
biuro@oilkrakow.org.pl
Tel: (4812) 6191720
Faks: (4812) 6191730

The assessment of the efficacy of different recovery strategies after fatiguing exercise

Background

Physical effort is always connected with fatigue, which decreases muscle power. Muscle fatigue is defined in terms of a reduction in the muscle power to perform action after physical effort has been made.The mechanisms responsible for the imbalance between fatigue and muscle recovery remain unclear. A wide range of recovery methods are now used as integral parts of the training programmes of elite athletes to help attain an optimal balance. Therefore it is crucial to determine their efficacy and substantiate their use.A variety of techniques and methods are used to increase the efficacy of post-exercise muscle recovery. There are however doubts about the influence of active recovery on subsequent performance, especially because in many studies there are methodological differences in relation to the task that is used as the performance criterion.

The aim of this study is the evaluation of different relaxation modes (active and passive) efficacy on muscle recovery. It is also assessed if application of various modalities of active recovery (work the same or different muscle groups than those, which were active during fatiguing exercise) allow us to observe significant differences in strength and bioelectrical activity of evaluated muscles, which may suggest that one of them is more effective in elimination of post exercise fatigue.

Material and Methods

Each subject will be invited to laboratory for 3 visits with one week intervals and for 1 pre-test visit for familiarisation. Each visit will begin with a warm-up, which consists of cycling for 5 minutes at a comfortable, self-selected speed. Measurements of the bioelectrical activity (EMG) of the vastus lateralis oblique

(VLO), the vastus medialis oblique (VMO), and the rectus femoris (RF) muscles and the torque, work and power of the knee flexor and extensor muscles will be performed during isokinetic testing at a velocity 90 degrees/s. The measurements will be perform before fatiguing exercise, after exercising and after one of the recovery modes. During pre-test visit the maximum velocity of each subject's treadmill running will be determined. The initial speed of the treadmill is 5 km/h and will be increased 1 km/h every 2 minutes, and physical effort will be performed to exhaustion, or to refusal to continue by the subject. From the maximal treadmill speed determined during pre-test visit the 120% will be calculated. The 100% it is the speed at the end of the effort when the subject is exhausted, but the 120% of this speed will be applicated during the effort when the subject will not be fatigued. The fatiguing exerccise will involve ten treadmill runs, each one-minute long (performed at the intensity of 120% of maximal treadmiil speed determined during pre-test visit) interspaced with two-minute breaks. During each two-minute break, the treadmill will be stopped and the subject will rest by standing on the treadmill. Then the treadmill will be started and accelerated to the 120% of maximal treadmill speed. The test physical effort will last 30 minutes (10 one-minute runs and 10 two-minute breaks). If the subject will not be able to complete the test at the required intensity, the velocity will be reduced by 0.5 km/h. Each of the recovery mode applied for 20 minutes immediately following completion of the fatiguing exercise. It will involve pedaling on the cycle ergometer (at a velocity of 60 rpm with a 10W load) pedaling on the arm ergometer (at a velocity of 60 rpm with a 10W load), or passive rest in a sitting position. The measurements will be performed directly before fatiguing exercise, immediately after exercising and after 20 minutes in one of the recovery modes. Each measurements will be performed by one physiotherapists during one-on-one consultation.
