# Supplementary material for: Comparison of Two Different Modes of Active Recovery on Muscles Performance after Fatiguing Exercise in Mountain Canoeist and Football Players
Source: PLoS One. 2016 Oct 5;11(10):e0164216. doi: 10.1371/journal.pone.0164216 (PMC5051742; doi:10.1371/journal.pone.0164216)
Supplement: S2 Protocol — (PDF) [file pone.0164216.s003.pdf]

Komisja Bioetyczna przy Okręgowej Izbie Lekarskiej w Krakowie
ul. Krupnicza 11a
31-123 Kraków, Polska
biuro@oilkrakow.org.pl
Tel: (4812) 6191720
Faks: (4812) 6191730

Analiza skuteczności różnych metod wspomagających relaksację mięśni po wywołujących zmęczenie ćwiczeniach fizycznych

Główne hipotezy badawcze – Cel badań

Trening sportowy nieodłącznie wiąże się ze zmęczeniem, które obniża możliwości pracy mięśniowej. Może ono utrzymywać się różnie długo i trwać kilka minut, godzin, a nawet utrzymywać się kilka dni. Brak właściwego wypoczynku sprawia, że zawodnicy nie mogą podejmować treningów z wymaganą intensywnością. Wysoki poziom zmęczenia jest również czynnikiem predysponującym do urazów. W celu zwiększenia skuteczności procesu powysiłkowej regeneracji sportowcy często stosują różne techniki i metody wspomagające ten proces. Jednym z kryteriów służących ocenie skuteczności danej metody wypoczynku jest to czy po jej zastosowaniu można zaobserwować szybszą regeneracje niż przy odpoczynku biernym. Mechanizmy odpowiedzialne za powstanie nierównowagi pomiędzy zmęczeniem i regeneracją mięśni oraz czynniki leżące u podstaw zmęczenia i wypoczynku nie są jednak do końca poznane. Obecnie w praktyce sportowej stosuje się wiele technik mających wspomagać proces regeneracji powysiłkowej. Dlatego niezwykle ważne jest, aby dokładnie poznać ich skuteczność i uzasadnienie ich użycia. Jakkolwiek większość badań wykazuje skuteczność wypoczynku czynnego w usuwaniu mleczanu z krwi, tak wpływ tej formy wspomagania relaksacji mięśni na zdolność do podjęcia kolejnego wysiłku nie jest już tak jednoznaczna. Niewiele jest bowiem prac opisujących np. zachowanie się siły mięśniowej po zastosowaniu różnych form wspomagania wypoczynku.

Celem badań jest analiza wpływu 3 różnych metod wspomagania powysiłkowej relaksacji mięśni na tempo powrotu ich sprawności przedwysiłkowej. Ocena skuteczności powysiłkowej relaksacji będzie przeprowadzona z uwzględnieniem poziomu sprawności fizycznej badanych, oraz w przypadku osób trenujących typowych dla danej dyscypliny sportu grup mięśniowych głównie obciążonych treningiem.

Postawiono następujące pytania badawcze:

1. Czy zastosowanie różnych form wypoczynku czynnego (praca tych samych lub innych grup mięśni niż te, które były aktywne podczas wysiłku testowego) pozwala zaobserwować istotne różnice w zakresie

parametrów będących wyznacznikiem skuteczności stosowanej formy wypoczynku?
2. Czy istnieją różnice pomiędzy poszczególnymi formami stosowanego wypoczynku czynnego, a wypoczynkiem biernym w tempie powysiłkowej relaksacji mięśni?
3. Czy istnieją różnice pomiędzy zastosowanymi formami wypoczynku czynnego w poszczególnych grupach badanych sportowców?

Materiał i metody badawcze

Badaniami objęte będą osoby w wieku 20-40 lat, niepalące, legitymujące się dobrym stanem zdrowia, bez schorzeń mogących mieć wpływ na przebieg i wynik badania.

Grupa 1 (n=30) - sportowcy trenujący regularnie – piłkarze nożni
Grupa 2 (n=30) – sportowcy trenujący regularnie – kajakarze górscy

Wszystkie pomiary zostaną przeprowadzone w godzinach przedpołudniowych aby zminimalizować wpływ dobowej zmienności bioelektrycznej aktywności mięśni. Każdy z badanych będzie uczestniczył w 4 spotkaniach przedzielonych każdorazowo tygodniową przerwą. Przed przystąpieniem do badań wszyscy uczestnicy zostaną szczegółowo poinformowani o ich przebiegu.

Wizyta wstępna
Podczas pierwszej wizyty uzyskane zostaną pomiary masy i wysokości ciała badanych. Po przeprowadzeniu rozgrzewki kolejno zostanie przeprowadzony familiaryzacyjny test izokinetyczny

Familiaryzacja do testu izokinetycznego - test ten służący do oceny czynnościowej mięśni zginaczy i prostowników działających na staw kolanowy wykonywany będzie w pozycji siedzącej z kończyną dolną zgiętą do kąta 90° w stawie kolanowym w ułożeniu, gdy oś obrotu będzie zgodna z osią anatomiczną stawu. Aby zapobiec ruchom tułowia podczas pomiaru siły badani przypięci będą pasem stabilizującym. W teście izokinetycznym podczas ruchu zgięcia i wyprostu w stawie kolanowym analizowane będą parametry siłowe i prędkościowe pracy mięśniowej przy prędkościach kątowych 90°/s. W każdym z testów badany wykona 10 naprzemiennych ruchów zgięcia i wyprostu w stawie kolanowym w maksymalnym zakresie ruchu. Zadaniem osoby badanej będzie wyzwolenie maksymalnej siły mięśniowej w jak najkrótszym czasie w każdym ruchu. Podczas pierwszej wizyty test izokinetyczny zostanie przeprowadzony w identyczny sposób jak podczas kolejnych wizyt, lecz uzyskane dane nie będą analizowane.

A. Ocena siły mięśniowej (test izokinetyczny) - będzie służył do oceny czynnościowej mięśni zginaczy i prostowników działających na staw kolanowy.

Parametrami siłowymi są:

- PT (peak torque - maksymalny moment siły) [Nm]
- MPT (peak torque/body mass – względny moment siły uwzględniający masę ciała badanego) [%]
- APT (average peak torque – średni moment siły) [Nm]
- TW (total work – praca całkowita określana jako całkowita suma momentów siły osiąganych podczas całego cyklu napięć izokinetycznych) [J]
- AP (average power – średnia moc) [W]

Parametrami prędkościowymi są:

- t_a (acceleration time, time to reach peak torque – czas osiągnięcia maksymalnego momentu siły) [ms]
- t_d (decceleration time – czas wyhamowania) [ms]

B. Ocena sEMG - parametry sEMG oceniające poziom zmęczenia i skuteczność relaksacji. Pomiar sEMG wykonywany będzie każdorazowo podczas testu izokinetycznego w warunkach dynamicznych. Oceniane będą mięśnie kończyny dolnej dominującej:VLO, VMO i RF. Analizowane będą parametry będące wyznacznikiem powysiłkowego zmęczenia mięśni i skuteczności zastosowanej relaksacji:

- parametry czasowe sygnału sEMG - RMS (root mean squere - parametr oceniający poziom aktywacji jednostek motorycznych podczas skurczu mięśniowego)
- parametry oparte na częstotliwości - F_m (mean frequency – zakres średniej częstotliwości całkowitego spektrum mocy – przesunięcie zakresu średniej częstotliwości w dół jest wyznacznikiem zmęczenia mięśnia)

C. Ocena intensywności wysiłku testowego - parametr określający maksymalną prędkość uzyskaną przez osobę badaną podczas biegu na bieżni. Prędkość początkowa bieżni to 5 km/h, i wzrasta o 1 km/h co 2 minuty, a wysiłek prowadzony jest do odmowy. Parametr ten będzie służył do określenia intensywności wysiłku testowego (120% z Vmax).

Wizyty 1,2,3

Kolejne wizyty będą służyły do oceny 3 różnych metod wspomagania relaksacji mięśni po intensywnych ćwiczeniach fizycznych.

A. Rozgrzewka

B. Test izokinetyczny z równoczesnym pomiarem sEMG - przed wysiłkiem (basenie)

C. Wysiłek testowy - bieg na bieżni 10 razy przez 1 minutę, każdy z intensywnością 120% Vmax z 2 minutową przerwą pomiędzy biegami. Gdy badany nie będzie w stanie wykonać całego testu (10 biegów) z wymaganą prędkością, prędkość będzie zmniejszana o 0.5 km/h. Podczas każdej 2 minutowej przerwy w biegu badany będzie odpoczywał biernie stojąc na bieżni. Prędkość początkowa bieżni to 5 km/h. Cały test wysiłkowy będzie trwał 30 minut 10x 1 min biegu i 10x 2 min przerwy

D. Test izokinetyczny z równoczesnym pomiarem sEMG - przeprowadzony będzie zaraz po zakończeniu wysiłku (pomiar poziomu zmęczenia)

E. Relaksacja - zaraz po zakończeniu testu izokinetycznego zastosowana zostanie jedna z trzech metod wspomagania relaksacji mięśni.
   - 20 minut jazdy na rowerze stacjonarnym z prędkością 60 obrotów/min i intensywnością 10 W
   - 20 minut jazdy na cykloergometrze ręcznym (praca kończyn górnych w celu pobudzenia innych grup mięśniowych niż te, które pracowały podczas wysiłku) z prędkością 60 obrotów/min i intensywnością 10 W.
   - 20 minut odpoczynku biernego w pozycji siedzącej

F. Test izokinetyczny z równoczesnym pomiarem EMG - przeprowadzony będzie zaraz po zakończeniu relaksacji (pomiar skuteczności relaksacji)
